# Bintrafusp Alfa: A Bifunctional Fusion Protein Targeting PD-L1 and TGF-β, in Patients with Pretreated Colorectal Cancer: Results from a Phase I Trial

**DOI:** 10.1093/oncolo/oyac254

**Published:** 2022-12-28

**Authors:** Alexander Spira, Michael S Wertheim, Edward J Kim, Benjamin Tan, Heinz-Josef Lenz, Petros Nikolinakos, Patricia L Rich, Genevieve Jehl, Andreas Machl, Rena Ito, James L Gulley, Scott Kopetz

**Affiliations:** US Oncology Research, Fairfax, VA, USA; Hematology Oncology Associates of the Treasure Coast, FL, USA; Department of Internal Medicine, UC Davis Comprehensive Cancer Center, Sacramento, CA, USA; Department of Medicine, Washington University School of Medicine, Siteman Cancer Center, St. Louis, MO, USA; Department of Medicine, Keck School of Medicine, University of Southern California Norris Comprehensive Cancer Center, Los Angeles, CA, USA; University Cancer & Blood Center, Athens, GA, USA; Cancer Treatment Centers of America, Southeastern Regional Medical Center, Newnan, GA, USA; Merck Healthcare KGaA, Darmstadt, Germany; EMD Serono Research & Development Institute, Billerica, MA, USAan affiliate of Merck KGaA; Merck Biopharma Co., Ltd., Tokyo, Japanan affiliate of Merck KGaA; Center for Cancer Research, National Cancer Institute, National Institutes of Health, Bethesda, MD, USA; Department of Gastrointestinal Medical Oncology, Division of Cancer Medicine, The University of Texas MD Anderson Cancer Center, Houston, TX, USA

**Keywords:** colorectal cancer, bintrafusp alfa, phase I

## Abstract

Colorectal cancer (CRC) is a heterogeneous and complex disease with limited treatment options. Targeting transforming growth factor β (TGF-β) and programmed death ligand 1 pathways may enhance antitumor efficacy. Bintrafusp alfa is a first-in-class bifunctional fusion protein composed of the extracellular domain of TGF-β receptor II (a TGF-β “trap”) fused to a human IgG1 monoclonal antibody blocking programmed cell death ligand 1. We report results from an expansion cohort of a phase I study (NCT02517398) in patients with heavily pretreated advanced CRC treated with bintrafusp alfa. As of May 15, 2020, 32 patients with advanced CRC had received bintrafusp alfa for a median duration of 7.1 weeks. The objective response rate was 3.1% and the disease control rate was 6.3% (1 partial response, 1 stable disease); 2 patients were not evaluable. The safety profile was consistent with previously reported data.

## Teaser

A phase I study of bintrafusp alfa showed early signs of clinical efficacy and a manageable safety profile in patients with heavily pretreated solid tumors.

## Introduction

Colorectal cancer (CRC) is a heterogeneous and complex disease with widely variable clinical outcomes.^1^ Although recent advances in systemic therapy for metastatic CRC (mCRC) have improved outcomes for patients with certain biomarkers, most patients receive chemotherapy ± bevacizumab, and the 5-year survival rate is only 14.7%.^2,3^ Programmed cell death 1 (PD-1)/programmed cell death ligand 1 (PD-L1) therapies have shown activity in patients with mismatch repair-deficient (dMMR) or microsatellite instability-high (MSI-H) CRC.^2^ However, patients with mismatch repair-proficient (pMMR) or microsatellite stable (MSS) CRC (80%-90% of patients) do not respond to PD-1 blockade alone.^1^ Limited activity may be due to mechanisms of resistance with PD-(L)1 and interplay with other pathways.^4,5^

Increased expression of transforming growth factor β (TGF-β) is associated with poor prognosis in CRC and contributes to the lack of response to PD-L1 blockade.^4,5^ Therefore, simultaneous inhibition of TGF-β and PD-L1 pathways may overcome anti-PD-(L)1 resistance and enhance antitumor efficacy in patients with CRC. Bintrafusp alfa is a first-in-class bifunctional fusion protein composed of the extracellular domain of the human TGF-β receptor II (a TGF-β “trap”) fused via a flexible linker to the C terminus of each heavy chain of an IgG1 antibody blocking PD-L1, which might allow for simultaneous inhibition of TGF-β and PD-L1 in the tumor microenvironment.^6^ In a phase I study (NCT02517398), bintrafusp alfa has shown early signs of clinical efficacy and a manageable safety profile in patients with heavily pretreated solid tumors.^7^ Here, we report results from an expansion cohort of this phase I study.

## Materials and Methods

In this open-label, global, phase I trial, adult patients with histologically confirmed advanced adenocarcinoma of the colon or rectum that progressed during or after second-line treatment, an Eastern Cooperative Oncology Group performance status of 0 or 1, and measurable disease per Response Evaluation Criteria in Solid Tumors (RECIST) version 1.1 were included. Patients must have had disease progression while receiving fluoropyrimidine, oxaliplatin, irinotecan, and bevacizumab, and cetuximab or panitumumab (for *RAS* wild-type tumors). Patient selection was not based on MSI, consensus molecular subtype (CMS), or PD-L1 status. Patients received bintrafusp alfa 1200 mg every 2 weeks until confirmed progressive disease (PD), unacceptable toxicity, or trial withdrawal.

The primary endpoint was confirmed best overall response (BOR) according to RECIST 1.1, as assessed by an independent review committee (IRC). The key secondary endpoint was safety, with adverse events coded according to Medical Dictionary for Regulatory Activities terms version 21.0 and classified by grade according to the National Cancer Institute Common Terminology Criteria for Adverse Events version 4.03. Exploratory endpoints included progression-free survival (PFS), overall survival (OS), and duration of response (Supplementary Methods S1).

## Results

As of May 15, 2020, 32 patients with advanced CRC had received bintrafusp alfa for a median duration of 7.1 (range, 2.0-100.0) weeks. Median follow-up was 185 (range, 3-185) weeks. All patients had discontinued treatment by the data cutoff. Overall, 87.5% of patients had  ≥ 3 prior anticancer therapies, and 81.3% had  < 1% tumor PD-L1 expression (Supplementary Table S1).

**Figure 1.**
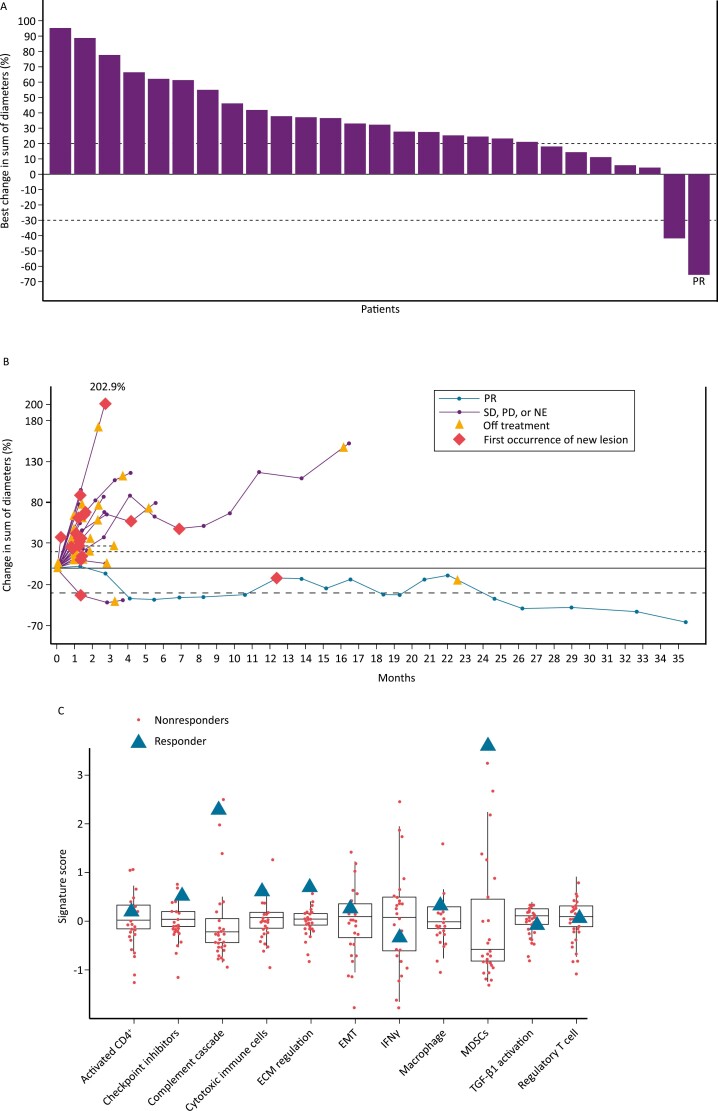
(**A**) Percent change in diameter from baseline in target lesions, as assessed by IRC per RECIST 1.1. (**B**) Percent change in target lesion diameter from baseline to best postbaseline value, as assessed by IRC per RECIST 1.1. (**C**) Patient gene signatures by RNAseq. The patient with a PR had colorectal cancer that was microsatellite stable, mesenchymal subtype (consensus molecular subtype 4), *KRAS* mutant, and PD-L1 positive (PD-L1 ≥ 1% tumor cell expression by immunohistochemistry). A higher signature score for complement cascade and myeloid-derived suppressor cells was observed. Of 32 patients who received bintrafusp alfa, 2 patients were not evaluable by IRC due to no IRC review (*n* = 1) and SD (or better) of insufficient duration (<6 weeks after start date without further evaluable tumor assessment) (*n* = 1). Abbreviations: IRC, independent review committee; RECIST, Response Evaluation Criteria in Solid Tumors; PR, partial response; SD, stable disease; PD; progressive disease; NE, not evaluable; ECM, extracellular matrix; EMT, epithelial to mesenchymal transition; IFNγ, interferon γ; MDSC, myeloid-derived suppressor cell; TGF-β, transforming growth factor β.

In the 30 evaluable patients, 1 had a confirmed partial response (PR), 1 had stable disease, and 28 had PD as BOR per IRC (Fig. 1A, B). The confirmed objective response rate (ORR) was 3.1% (95% CI, 0.1-16.2), with a disease control rate (DCR) of 6.3% (95% CI, 0.8-20.8) per IRC. Median PFS was 1.3 (95% CI, 1.2-1.4) months (Supplementary Fig. S1); median OS was 7.7 (95% CI, 3.7-14.0) months (Supplementary Fig. S2). The patient with a PR had a fresh biopsy taken within 7 days before treatment start and was found to have *KRAS* mutant MSS CRC and CRC consensus molecular subtype 4 (CMS4), the poor-prognosis mesenchymal subtype.^8^ Although this subtype is associated with TGF-β activation, this was not observed (Fig. 1C). Interestingly, however, the patient had a higher signature score for complement cascade and myeloid-derived suppressor cells (Fig. 1C), indicative of inflammation and immunosuppression in the CMS4 subtype.^8^ This patient had the highest tumor cell PD-L1 expression (20%) amongst the 32 patients, completed 100 weeks of treatment with bintrafusp alfa, and had a duration of response of 8.3 months.

Any grade treatment-related adverse events (TRAEs) occurred in 68.8% of patients; grade 3 occurred in 12.5% (Table 1). The most common TRAEs of any grade were nausea (18.8%), anemia (15.6%), diarrhea (15.6%), and infusion-related reaction (15.6%). No grade 4 TRAEs or treatment-related deaths were reported. One patient had grade 3 enteritis that co-occurred with disease progression, leading to permanent treatment discontinuation.

**Table 1.** Treatment-related adverse events.

	Bintrafusp alfa, n (%) *N* = 32	
	Any grade	Grade 3
Any TRAE	22 (68.8)	4 (12.5)
Nausea	6 (18.8)	0
Anemia	5 (15.6)	1 (3.1)
Diarrhea	5 (15.6)	0
Infusion-related reaction	5 (15.6)	0
Decreased appetite	4 (12.5)	0
Fatigue	4 (12.5)	1 (3.1)
Myalgia	3 (9.4)	0
Pyrexia	3 (9.4)	0
Rash	3 (9.4)	0
Vomiting	3 (9.4)	0
Abdominal pain	2 (6.3)	0
Dermatitis acneiform	2 (6.3)	0
Malaise	2 (6.3)	0
Rash maculopapular	2 (6.3)	0
Stomatitis	2 (6.3)	0
Adrenal insufficiency	1 (3.1)	1 (3.1)
Blood bilirubin increased	1 (3.1)	1 (3.1)
Enteritis	1 (3.1)	1 (3.1)

## Discussion

There is a high unmet need for treating MSS metastatic CRC. In phase I study, MSI testing was performed on 50% of enrolled patients, all of whom had MSS CRC. A single PR (duration of 8.3 months) was observed. The ORR was 3.1% per IRC assessment, with a DCR of 6.3%. Current studies of anti–PD-1 agents suggest that their clinical effect is generally unencouraging in patients with pMMR or MSS CRC.^9,10^ The observed modest antitumor activity of dual inhibition in the present study suggests that simultaneous inhibition of TGF-β and PD-L1 pathways with bintrafusp alfa could not overcome PD-L1 resistance across this patient population. Based on the gene signature profile of the patient who showed a partial response, it is possible that a pre-selected population could benefit from treatment with this dual-targeted immunotherapy, potentially those selected for CMS4 and PD-L1 status. Selection for CMS4 CRC and high PD-L1 status should be tested further in additional trials.

Grade 3 TRAEs occurred in 4 patients (12.5%), and no grade ≥4 TRAEs or treatment-related deaths occurred. Bintrafusp alfa had a manageable safety profile in patients with advanced CRC, consistent with that observed in patients with solid tumors^7^ and with what is expected with dual inhibition of TGF-β and PD-L1.

Study limitations include the lack of a comparator arm and the small number of patients, which made it difficult to interpret the magnitude of benefit with bintrafusp alfa. The findings of this study suggest that bintrafusp alfa may play a role in tumor control in select patients; however, further investigation is needed.

## Conclusion

Dual inhibition of TGF-β and PD-L1 by bintrafusp alfa demonstrated modest antitumor activity and a manageable safety profile in patients with heavily pretreated, advanced CRC. Further studies are needed to better understand the role of TGF-β and PD-L1 in advanced CRC and identify potential subsets of patients who may benefit from dual inhibition of these pathways.

## Supplementary Material

oyac254_suppl_Supplementary_Figures

oyac254_suppl_Supplementary_Material

oyac254_suppl_Supplementary_Table

## Data Availability

Any requests for data by qualified scientific and medical researchers for legitimate research purposes will be subject to Merck’s Data Sharing Policy. All requests should be submitted in writing to Merck’s data sharing portal (https://www.merckgroup.com/en/research/our-approach-to-research-and-development/healthcare/clinical-trials/commitment-responsible-data-sharing.html). When Merck has a co-research, co-development, or co-marketing or co-promotion agreement, or when the product has been out-licensed, the responsibility for disclosure might be dependent on the agreement between parties. Under these circumstances, Merck will endeavor to gain agreement to share data in response to requests.
