# Supplementary material for: Bintrafusp Alfa: A Bifunctional Fusion Protein Targeting PD-L1 and TGF-β, in Patients with Pretreated Colorectal Cancer: Results from a Phase I Trial
Source: Oncologist. 2022 Dec 28;28(2):e124–7. doi: 10.1093/oncolo/oyac254 (PMC9907041; doi:10.1093/oncolo/oyac254)
Supplement: oyac254_suppl_Supplementary_Material [file oyac254_suppl_supplementary_material.docx]

**SUPPLEMENTAL MATERIAL**

**Methods**

Patients were required to have tumor archival material or fresh biopsy specimens within 28 days of the first administration of bintrafusp alfa, and all biomarker testing was performed at the local investigator site (or central laboratory if local testing is not available). While PD-L1 analysis was performed in a CLIA compliant lab, mRNA sequencing was done in an exploratory fashion and was not CLIA compliant. CMS was computed using the CMSclassifier R package (https://github.com/Sage-Bionetworks/CMSclassifier) that was published previously.^8^ The classifier was run on log2 transformed TPMs as produced by RSEM using Ensembl transcripts and the hg19 genome. The MSS/MSI data was generated at local sites and may or may not have been CLIA-compliant. Tumor cell programmed cell death ligand 1 (PD-L1) expression was assessed centrally with a proprietary immunohistochemistry assay (Dako 73-10). PD-L1 positivity was defined by a threshold level of ≥1% PD-L1–positive tumor cells of any intensity. To obtain the signature score for a given gene expression signature in a sample using RNAseq, the log_2_ of transcripts per million (TPM) for each gene in the sample was determined and then normalized by subtracting the mean log_2_ TPM across all samples. The signature score for a given signature in a sample is the mean of the normalized log_2_ TPMs for each gene in the signature.

**Statistical plan**

With a planned enrollment of 30 patients, the study was designed to have 79% power to reject the null hypothesis of an objective response rate (ORR) of ≤10%. The sample size in this cohort was a practical number in order to obtain preliminary estimates of efficacy. The primary endpoint, ORR, was determined as the proportion of patients with a confirmed best overall response (complete response or partial response). The 95% exact (Clopper-Pearson) confidence intervals for ORR based on 30 enrolled subjects were calculated to provide precision of estimated response rates based on a total sample size of 30 subjects under different assumptions on the true response rate in the overall population. Descriptive statistics were used to analyze safety.
