# Supplementary material for: Bintrafusp Alfa: A Bifunctional Fusion Protein Targeting PD-L1 and TGF-β, in Patients with Pretreated Colorectal Cancer: Results from a Phase I Trial
Source: Oncologist. 2022 Dec 28;28(2):e124–7. doi: 10.1093/oncolo/oyac254 (PMC9907041; doi:10.1093/oncolo/oyac254)
Supplement: oyac254_suppl_Supplementary_Table [file oyac254_suppl_supplementary_table.docx]

**Table S1.** Baseline Characteristics in CRC Expansion Cohort

| **Characteristic** | **Bintrafusp alfa, n (%) N = 32** |
| --- | --- |
| Sex | |
| Male | 16 (50.0) |
| Female | 16 (50.0) |
| Age, years | |
| <65 | 21 (65.6) |
| >65 | 11 (34.4) |
| ECOG performance status | |
| 0 | 11 (34.4) |
| 1 | 21 (65.6) |
| Tumor PD-L1 expression^a^ | |
| >1% | 3 (9.4) |
| <1% | 26 (81.3) |
| Unknown | 3 (9.4) |
| Prior anticancer regimens | |
| 2 | 4 (12.5) |
| 3 | 9 (28.1) |
| ≥4 | 19 (59.4) |
| *KRAS* mutational status | |
| Wild type | 11 (34.4) |
| Mutant | 21 (65.6) |
| Mismatch repair status | |
| MSS | 16 (50.0) |
| MSI-H | 0 (0.0) |
| MSI-L | 0 (0.0) |
| Unknown/missing | 16 (50.0) |
| Primary tumor type | |
| Adenocarcinoma of the colon | 23 (71.9) |
| Adenocarcinoma of the rectum | 9 (28.1) |
| Tumor sidedness^b^ | |
| Left | 13 (40.6) |
| Right | 9 (28.1) |
| Not evaluable | 10 (31.3) |
| Type of previous anticancer treatment^c^ | |
| Cytotoxic | 32 (100.0) |
| Monoclonal antibodies | 31 (96.9) |
| Small molecules | 14 (43.8) |
| Other^d^ | 5 (15.6) |

^a^PD-L1 positivity was defined by a threshold level of ≥1% positive tumor cells of any intensity detected by immunohistochemistry (IHC) using a proprietary assay (Dako PD-L1 IHC 73-10 pharmDx [Dako, Carpinteria, CA, USA]).

^b^Tumor sidedness was not prospectively collected but assigned based on patient’s prior listed cancer surgery.

^c^Treatment for locally advanced or metastatic disease categorized per investigator’s discretion.

^d^Determined by investigator to be not a “cytotoxic therapy”, “monoclonal antibody therapy” or “small molecule”.

Abbreviations: ECOG, Eastern Cooperative Oncology Group; EGFR, epidermal growth factor receptor; MSI-H, microsatellite instability-high; MSI-L, microsatellite instability-low; MSS, microsatellite stable; PD-L1, programmed cell death ligand 1; VEGFR, vascular endothelial growth factor.
